# Landscape Analysis of Drone Congregation Areas of the Honey Bee, *Apis mellifera*

**DOI:** 10.1673/031.012.12201

**Published:** 2012-10-22

**Authors:** Alberto Galindo-Cardona, A. Carolina Monmany, Rafiné Moreno-Jackson, Carlos Rivera-Rivera, Carlos Huertas-Dones, Laura Caicedo-Quiroga, Tugrul Giray

**Affiliations:** Department of Biology, University of Puerto Rico, JGD 202 PO Box. San Juan, PR 00931

**Keywords:** DCAs, landscape ecology, mating behavior, queen pheromone

## Abstract

Male honey bees fly and gather at Drone Congregation Areas (DCAs), where drones and queens mate in flight. DCAs occur in places with presumably characteristic features. Using previously described landscape characteristics and observations on flight direction of drones in nearby apiaries, 36 candidate locations were chosen across the main island of Puerto Rico. At these locations, the presence or absence of DCAs was tested by lifting a helium balloon equipped with queen-sex-pheromone-impregnated bait, and visually determining the presence of high numbers of drones. Because of the wide distribution of honey bees in Puerto Rico, it was expected that most of the potential DCAs would be used as such by drones and queens from nearby colonies. Eight DCAs were found in the 36 candidate locations. Locations with and without DCAs were compared in a landscape analysis including characteristics that were described to be associated with DCAs and others. Aspect (direction of slope) and density of trails were found to be significantly associated with the presence of DCAs.

## Introduction

Studying landscape properties of drone congregation areas (DCAs) is important both for understanding bee behavior, and because such areas have practical uses for genetically controlled mating in bee breeding programs and to delimit conservation areas for subspecies of honey bees (Ruttner 1976). In addition, studies on DCAs of honey bees are of increased relevance given the recent concerns about decreasing numbers and affected health of these generalist pollinators (Oldroyd 2007; Aizen and Harder 2009; Giray et al. 2010; Neuman and Carreck 2010; Mullin et al. 2010). DCAs of the honey bee *Apis mellifera* L. are places outside the colony where hundreds of males and reproductive females (“queens”) assemble, especially in afternoon hours. Bees mate in flight, and there have been reports of mating events taking place at heights ranging from 15 to 60 m, with the flying bees reaching velocities of up to 12 kilometers (km) per hour (Oertel 1956; Zmarlicki and Morse 1963; Loper et al. 1992). Although not all mating events take place at DCAs, and some occur on the flight paths of drones (Koeniger et al. 2005), these congregations appear to be important for honey bee reproductive behavior. Bee colonies in an area are faithful to DCAs, and the visits appear not to be limited by experience, since different generations of drones from the colonies frequent the same sites every mating season, and unmated queens also arrive at these locations (Laidlaw and Page 1984; Schlüns et al. 2005).

The factors attracting drones and queens to DCAs are not fully known, but one hypothesis involves specific physical (landscape) characteristics of the areas (Winston 1987). Here, we refer to this as the “physical DCA hypothesis.” Although it is known that prominent geographical landmarks play an important role in drone orientation (Zmarlicki and Morse 1963), no analysis has been done on the landscape characteristics of these places using Geographic Information Systems (GIS). Distance is another important physical property, with a negative relationship between DCA distance to the apiary of origin and drone visitation rates, presumably due to energy expense and vulnerability to predators (Koeniger et al. 2005). The DCAs are typically observed at a distance of 500 m to 5 km from the bee colony (Ruttner 1976). An alternative hypothesis is the “behavioral DCA hypothesis,” which says that DCAs could result from behavioral interactions of flying drones and queens (Loper et al. 1992). The physical and behavioral DCA hypotheses are not mutually exclusive. It is possible that certain flight paths with particular characteristics lead to particular interactions.

Finding and studying DCAs is important for studies on animal navigation, conservation, and population genetics. Patience and long searches, combined with the use of insect radars, has allowed the finding of several DCAs, and the mapping of flights around these DCAs (e.g. Loper et al. 1992). Understanding geographical and other characteristics of DCAs could help develop an easier way to find a larger number of these areas using the same pheromone-assisted search methods. Identifying even a few DCAs facilitates the estimation of genetic diversity and genetic structure (i.e., Collet et al. 2009). Currently, the health of bees can be monitored using gene expression or microbiological analysis (Evans 2006; Robinson et al. 2008) on worker bees collected from colonies in a sampling area. It is, therefore, possible to use male bees at a DCA as a sample of genetically distinct colonies in the area and examine, at the least, prevalence of disease organisms or stress and immune gene expression in these male bees. Defining mating behavior is a very important tool for developing strategies to preserve the genetic variation found in the native habitats of the bee (Strange 2004; Collet et al. 2009), helping to maintain resilient agricultural bee populations.

Our purpose here is to compare the landscape characteristics of the locations where DCAs are present with those where DCAs are absent in Puerto Rico in order to test predictions of the physical and behavioral DCA hypotheses. It is expected that the descriptions of DCAs will help in finding new DCAs on the island and elsewhere.

## Materials and Methods

### Sampling

**Figure 1.**
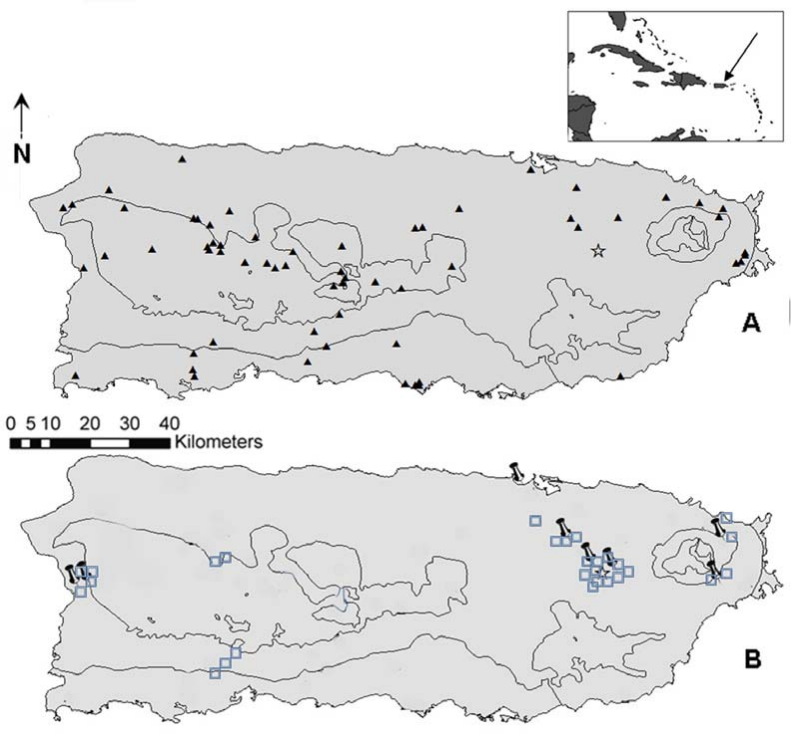
Map of Puerto Rico showing A. apiaries (black triangles), and our apiary (star), and B. areas where Drone Congregation Areas were present (pin point, n = 8) and absent (blue squares, n = 28). High quality figures are available online.

In Puerto Rico, 70 apiaries were geographically referenced using a Global Positioning System. In order to represent the geographic diversity of the island (east to west, high to low, central to coastal regions, in dry and wet climate areas; see Rivera-Marchand et al. in press), 14 out of 70 apiaries were selected and utilized as reference points for DCA searches. In total, 36 sites near these apiaries were classified in this study during the mating seasons (May-October) of years 2008 and 2009 (Figure 1). Eight out of 36 were DCAs, and 28 were not. From previous experiments, it is known that drones in Puerto Rico exit to fly between 14:00 and 17:30, and are regularly able to fly back and forth to locations at a 2 km radius (Galindo-Cardona 2010). Using this information as a guide, the DCA candidate locations in this study were sampled within a 2 km distance from known reference apiaries.

Features such as rivers, trail intersections, forest gaps, and large objects such as big trees have been found to be important for directing drones and queens in their flight (Ruttner 1976; Loper et al. 1992). Therefore, potential DCAs were expected to be found near such potential navigational aids to bees. Field observations confirmed that DCAs lie in open areas (pastures) surrounded by trees or high vegetation, apparently providing an open place where the mating events can take place, while still offering shelter from wind. Places in accordance with this description were located close to known apiaries and in the general flight direction of drones (which was previously determined by field observations), marked as potential DCAs using Google Earth®, and then visited in the field. At each potential DCA, a kite or a helium balloon was raised to 30–45 meters (m) above ground, at a 200 m to 2 km distance from the reference apiary during the drone flight time (14:00 to 17:30). The kite or balloon carried Flexlures (Contech Enterprises Inc., www.contechinc.com) queen mandibular pheromone (9-hydroxy-2-enoic acid) as bait (Williams 1987). Zmarlicki and Morse (1963) used only 9-oxodec-2-enoic acid. Observations were made at colony entrances, and vanishing bearings (Capaldi et al. 2000) of male bees exiting three different colonies were recorded. This helped determine the general flight direction of drones for that apiary. With the exception of two apiaries, drones departed only in one general direction. In the apiaries where drones seemed to depart in different directions, multiple DCAs were found. The drone orientation and flight will be discussed in a separate manuscript (Galindo-Cardona, Giray and colleagues, unpublished results). The search was started at approximately 200 m from a reference apiary, raising the balloon and bait in order to determine the return flight path of the drones near the vanishing bearing of males. Once a drone flight path was found, the bait was moved in this direction away from the apiary in a straight line. During this process, at every 200 m the bait was moved 200 m to each side of the centerline, in a zigzag search pattern. The pheromone bait was raised at preselected open areas, as described above, on or near the drone flight path for up to a distance of 2 km from the colony.

An area was defined as a DCA if drones were present in large numbers, and confirmed by taking pictures and videos of the drone groups and of the comets that drones formed (at times reaching numbers over 1,000 individuals, as in Loper et al. 1992). Then, coordinates of the locality were taken, and it was noted whether or not it was a DCA (Supplemental Table). A DCA was deemed stable if numerous drones were found there on at least two nonconsecutive days (separated at least by two weeks). Two of these DCAs were close to two different reference apiaries, and six were each near one reference apiary (Figure 1). These sites were found to have aerial boundaries, as reported in previous studies (Ruttner and Ruttner 1965), because the pheromone was ignored by drones just a few meters outside the DCA. DCAs were not found in 28 other locations close to the same reference apiaries and on or near drone flight paths.

### Environmental variables

Each georeferenced site with DCAs (n = 8) and without DCAs (n = 28) was placed over high definition digital aerial photographs (resolution: 0.33 m), and analyzed using Geographical Information System software (ArcGIS v. 9.3, Esri Inc., www.esri.com/software/arcgis) to map and retrieve geographical data from shapefiles (files that contained the information of landscape and environmental characteristics of the tested sites). Four concentric circular buffer areas were delineated and centered on each sample site with radii of 100, 200, 400, and 800 meters (supplemental Figure). The buffer was the unit area that ArcGIS used to analyze different layers of spatial information. On each site, the following land cover types were measured: pasture, urban (an area covered by buildings or paved surfaces), and crops (Gould et al. 2008). The Digital Elevation Model (DEM) is a computer representation of the earth's surface, and as such, provides a data base set from which topographic parameters can be digitally generated. The highest resolution DEM available in Puerto Rico (5 × 5 meters) was used to calculate the slope, aspect, and average solar radiation during mating season within each buffer. Aspect is the compass direction toward which a slope faces. Aspect could influence wind direction, vegetation growth, and solar radiation, among other factors. Aspect information can be generated from continuous elevation surfaces (ESRI, ArcGIS® v. 9.3.). The solar radiation data during mating season was calculated based on the DEM. Solar radiation is important because honey bees use the sun for orientation in flight (von Frisch 1967; Winston 1987). The numbers of apiaries, rivers, and trails were digitalized from high-definition aerial photographs, and added to the database for analyses using ArcGIS.

### Data analysis

Each of the four circular buffer radii was described according to: percent cover of pasture, urban, and crops; mean and standard deviation of solar radiation; aspect; slope (McCune and Keon 2002); and number and density of apiaries, rivers and trails. In order to estimate direct incident solar radiation based on slope and aspect, radians were used. These variables were placed in a matrix with the other landscape variables. The transformation to radians was necessary because 1° is adjacent to 360°. As in this example, the numbers may be very different even though the aspect would be practically identical (McCune and Keon 2002). Multi-Response Permutation Procedure (MRPP) was used to examine the differences between DCAs and non- DCAs in terms of the mentioned landscape characteristics across the four groups or buffer radius distances (100, 200, 400 and 800 m) (Supplementary Figure). MRPP is a nonparametric procedure for testing the hypothesis of no difference between two or more groups of entities (McCune and Mefford 1999). It is a practical technique, as it does not require assumptions such as normality and equal variance (Biondini et al. 1988). The within-group chance-corrected agreement (*A-value*) has a maximum of 1 when there is within-group homogeneity compared to the random expectation. The *P*-value is the probability of obtaining, by chance, a value of *A* equal to or larger than the observed value. In PC-ORD 5.0 (McCune and Mefford 1999), the MRPP tests were run using Euclidean distances, which measure differences using the Pythagorean theorem to N dimensions; it is the most conservative measure of distance (McCune et al. 2002).

**Supplemental Table.**
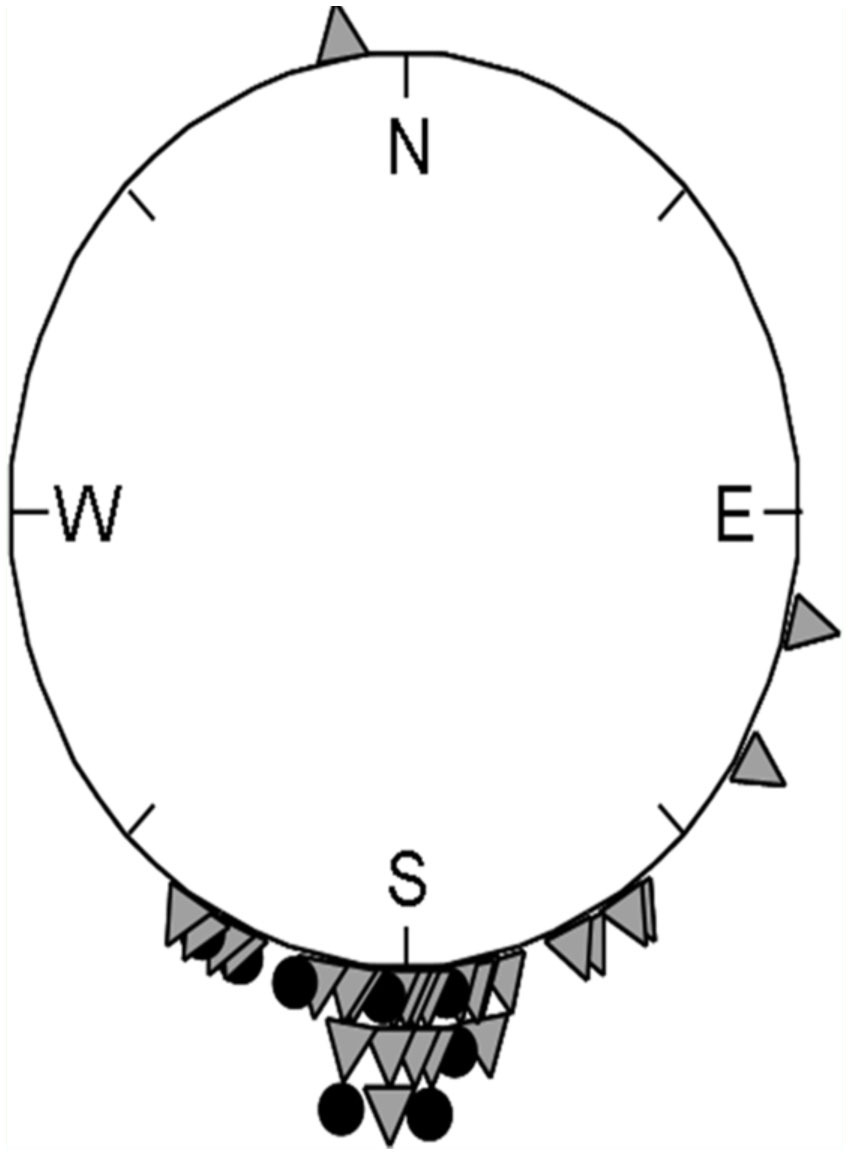
Variables used in the MRPP and the Partition Analysis to examine landscape effects on the location of Drone Congregation Areas. Value, Class name, Unity of measure and range are shown. Fourteen landscape categories were analyzed (C1 to C14), solar radiation (SR-), aspect (ASP-), slope (SLO-), and density of tracks (Track-). Abbreviations are as follows: Pres-Abs = presence (DCA)-absence (NoDCA) of DCA; Dist = radii of distance buffers; SR-M = Solar Radiation -Mean SR-STD = Solar Radiation -Standard Deviation; ASP-M = Aspect -Mean; ASP-F = Folded Aspect; ASP-R = Aspect in Radians; SLO-M = Slope -Mean; SLO-R = Slope in Radians; Tracks-M = Density of tracks and trails- Mean; Tracks-STD = Density of tracks and trails- Standard Deviation.

**Figure 2.**
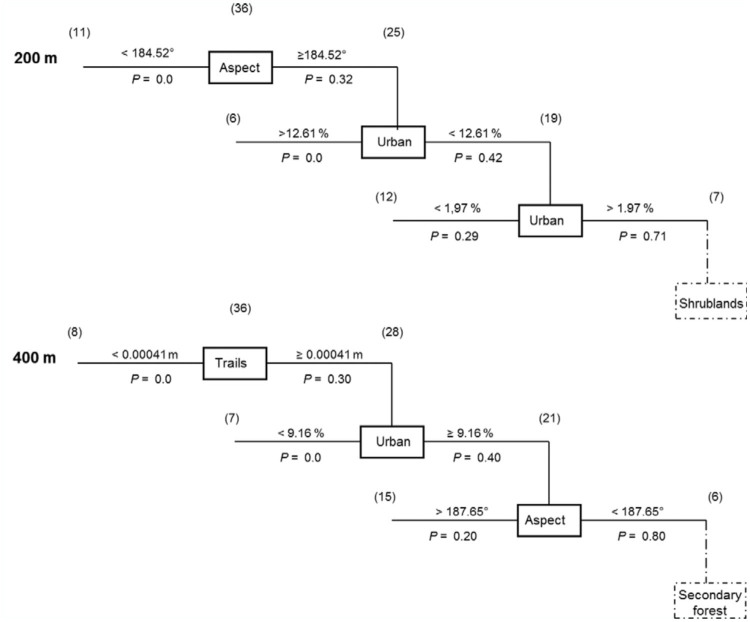
A Recursive partitioning analysis separated eight DCAs from the other 28 locations at 200 and 400 m. The tree-view shows the significant landscape characteristic in the box, the number of locations on the trunk and the number of separated sites in parentheses. The cut off values are above the line and the *P* values are below the line. *P* value indicates the probability of randomly finding a DCA with these landscape characteristics. The analysis was stopped after three layers (shown in solid lines) of partitioning (see Segal 1995). Dashed lines show less significant landscape characteristics. High quality figures are available online.

Recursive Partitioning Analysis (De'ath and Fabricius 2000) was used in JMP™ version 8 to discriminate the most correlated landscape characteristics of a site with the formation of a DCA. This analysis uses a classification tree to explain a single response variable that can be categorical or numeric, to create splits that result in more homogeneous groups (De'ath and Fabricius 2000; Quinn and Keogh 2002). The model divides a dataset by selecting the single variable that accounts for the most variability between two groups (DCA and non-DCA), and makes a split in the dataset using that particular variable. This process is then repeated on each of the two groups, resulting from the previous split, with each variable being assessed at every split whether the variable was previously used or not (Rejwan et al. 1999). This analysis is an exploratory approach that creates a regression tree according to significance among the variables. The groups are continually split into more homogeneous groups, theoretically, until only a single location remains as a group, or until there is no variation between locations in a group (Clark and Pregibon 1992). Conservatively, the recursive partitioning is carried out for three levels, or until there are only five or fewer individuals in one of the split groups (see also Giray et al. 2010). Two buffer radii (200 and 400 m) determined the most important landscape variables for classifying a DCA (Figure 2). In addition, homogeneity of multiple circular variances of aspect was tested using a Bartlett's Chi-square test (Zar 1999).

## Results

The MRPP analysis revealed differences in landscape characteristics among the different buffer radius distances (*A* = 0.02, *P* = 0.005, n = 4), and a trend in both presence/absence of a DCA (*A* = 0.008, *P* = 0.06, n = 2). However, these analyses do not identify which landscape characteristics are important for the DCA formation. To identify landscape characteristics important for DCA presence, a Recursive Partitioning Analysis (RPA) was performed.

In buffer areas with radii of 200 m (R^2^ = 0.53) and 400 m (R^2^ = 0.56), aspect or orientation of the terrain, percent urban cover, and density of trails explained the presence of DCAs (Figure 2). The tests showed that the probability that locations with a combination of aspect and urban cover in the 200 m radius contain a DCA is 71 %. If any other variable was added to the model, this probability fell drastically. Similarly, at 400 m, the most important variables were trails, urban cover, and aspect. The combination of all three predicted 80% of the locations containing a DCA. This probability fell when a different characteristic was considered. For instance, it fell to 33% when secondary forest cover was added. Densities of linear marks such as trails are very low in the DCA locations, indicating that perhaps one or a few trails crossed the area with a DCA (Figure 2). In contrast, the RPA found that at 100 m (R^2^ = 0.34) and 800 m (R^2^ = 0.29), distinguishing the areas where DCAs were present or absent was not possible; this means that a strong correlation among the landscape variables and presence-absence of DCAs was not found in these zones (see Discussion).

**Figure 3.**
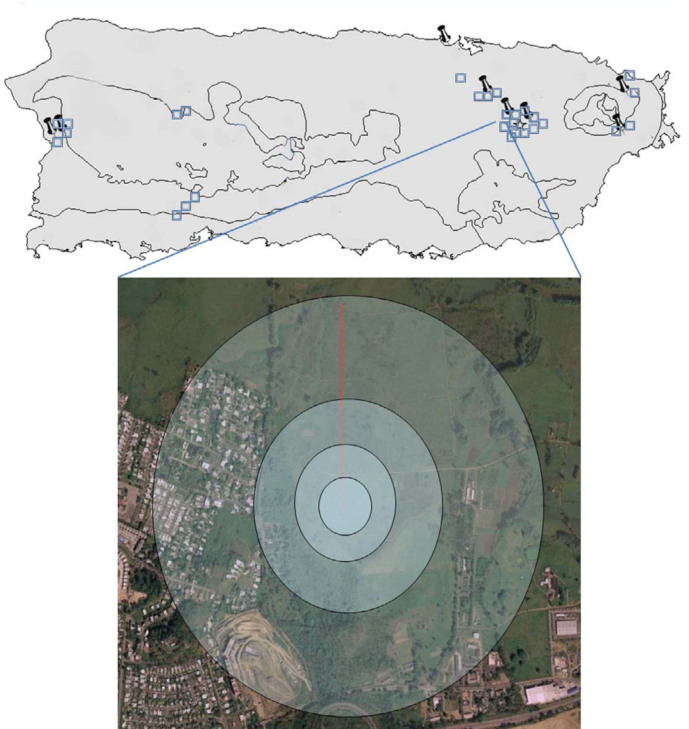
Aspect of locations of DCAs (circles) and locations of non DCAs (triangles) at 400 meters (0 = N, 90 = E, 180 = S, 270 = W) in a circular distribution relative to the elevation of the landscape. Most locations are at a similar direction because of selection based on drone flight direction from the colonies, however, locations with DCAs are significantly more concentrated facing the South (see statistics in the results). At 800 meters we also found significant differences between DCA and Non-DCA locations. High quality figures are available online.

In RPA, aspect was correlated with presence of DCAs in the 200, 400, and 800 m buffer zones. This variable was further examined in a separate analysis. Barlett's chi-square tests for circular variances showed that areas with DCAs had lower variance in aspect than areas without DCAs. Differences were statistically significant at 400 m (circular variance, *S*_DCA_ = 0.023; *S*_non-DCA_ = 0.152; *X*^2^ = 6.58, df = 1, *P* < 0.01) (Figure 3); and 800 m (*S*_DCA_ = 0.026; *S*_non-DCA_ = 0.102; *X*^2^ = 3.8, df = 1, *P* < 0.05) but not at 200 m (*P* = 0.11).

## Discussion

A limited set of physical characteristics of the landscape was found to correlate with the presence of DCAs at 200 and 400 m radius centered on the congregation area, supporting the physical DCA hypothesis. Aspect consistently was among the most important correlates of presence of a DCA in a candidate location (see Figure 2). These factors have been previously suggested to be important for DCAs (Zmarlicki and Morse 1963), yet this is the first study that tests the hypothesis comparing DCA and non-DCA locations in a GIS analysis.

Significant differences were not found in landscape characteristics between DCAs and non-DCAs at 100 m and 800 m radii. This is most likely due to a methodological issue. At 100 m radius, characteristics reported in literature were used to search for the DCAs and elevate the pheromone bait, which may have resulted in a higher homogeneity in the landscape of the areas at this radius between DCAs and non-DCAs. In contrast, at 800 m, a large physical overlap was observed across DCA and non-DCA as a function of greater radius. However, two previous studies (Capaldi et al. 2000; Menzel et al. 2001) found that worker bees reach a distance of 300 m from the hive in orientation flights when they first depart from the colony. Based on these studies, one hypothesis would be that drones are able to better detect features of the landscape within a similar range (between 200 and 400 m) when flying at or around the DCAs.

At virtually all buffer distances, non-DCA sites showed higher aspect variance than DCA sites (Figure 3). The mean aspect values of areas around DCAs clustered with those of non-DCAs, probably because only areas in the general return path of drones were searched. Yet, the localities with DCAs had aspects more concentrated towards the South than locations where no DCAs were encountered. Indeed, statistically significant differences were found in buffer areas of both 400 and 800 m where DCAs exhibited a significantly more southern aspect (see Figure 3). At these distances, the variances for aspect for areas with DCA vs. non-DCAs were not equal. DCAs had a variance closer to 0, caused by a more clumped distribution towards the mean direction.

**Figure 4.**
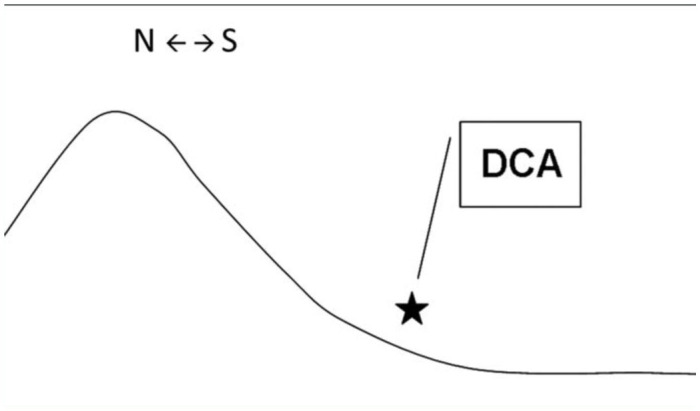
Diagram of a topographical cross-section (side view) of a slope with a southern aspect. The star indicates the location of a Drone Congregation Area. N ← → S represents North and South. High quality figures are available online.

The southern aspect of DCAs could be particular to Puerto Rico; however, in any location, a general preferred aspect of land features that mark DCAs would allow drones flying from different colonies to converge. The aspect may then serve as an orientation cue used by reproductive honey bees from different colonies in order to converge on a congregation area (Figure 4). Such a navigational adaptation would be similar to that observed in migratory birds and insects, where all individuals fly in one preferred direction to converge on reproductive areas, relatively independent of how far to east or west of the target they start (Wehner and Wehner 1990; Berthold and Pulido 1994). Based on starting locations, the migratory insects may be taking flights in different directions, but this results in reaching the same target destination (Wehner and Wehner 1990; Brower 1996). Several studies have shown that insects combine map and compass sense to go in the right direction (Merlin et al. 2011). In a bumble bee study, bees were shown to choose the north or south (as opposed to the east or west) for approach to land at the nest. This orientation choice is determined by the distribution of light, the wind direction, and the skyline (Hempel de Ibarra et al. 2009).

One hypothesis to explain the south aspect as preferred for DCAs is the earth's geomagnetism that is probably used by drones for orientation. The values of geomagnetism are different across the landscape, and honey bees have magnetic sense orientation. There is evidence of magnetic sense in honey bees both behaviorally and anatomically (Hsu et al. 2007, and references therein). The following hypothesis can be tested in future studies: Can bees tell the north or south side of the landmark or hill according to magnetoreception mechanism, as hypothesized by Hsu et al. (2007)? There is also new information connecting circadian rhythm, light sensitivity, and magnetic sense in insects through molecular substrates (Yoshi et al. 2009). Drone flight is a honey bee behavior that shows a strong circadian pattern and involves important navigational abilities. It would be possible to test the importance of different cues for selection of mating areas by honey bees by experimentally targeting their circadian rhythm and its potential interacting mechanisms, such as magnetic sense, using the increasingly varied pharmacological and molecular toolbox of honey bee behavioral research (Rubin et al. 2006; Robinson et al. 2008).

Alternatively, other factors may contribute to the south aspect of DCAs in Puerto Rico. One such factor is the distribution of light. The sunlight in temperate and tropical zones is important for geographical orientation. Many tropical bee species, including *A. mellifera*, place the nest entrance pointing south (Winston 1987; Soucy et al. 2003). In addition, there is a mechanistic explanation for determining a southerly direction in relation to the sun. This explanation is that bees use the sun as a compass for orientation (von Frisch 1967). One caveat is that, although in tropical zones the direct southern aspect has more hours of sun exposure, southeast should be preferred in the temperate areas based on the sunlight criterion (McCune and Keon 2002). However, in previously published studies of large DCAs, similar to those in this study, authors also found a southern aspect (Lindauer 1985; Ruttner 1985; Loper et al. 1992).

Yet another factor is wind direction. Wind is an important factor for mating bees; copulation takes place in flight, and the range of the queen sex pheromone is affected by wind speed and direction. The main air currents in Puerto Rico are the Trade Winds, which usually blow from the northeast, and winds from the Caribbean Sea, usually from a southeastern direction, so a northwestern aspect for DCAs would be predicted if protection from the wind were the most important factor. Indeed, it is found that orchids on tree trunks in Puerto Rico demonstrate a northwest location, avoiding the northeast and southeast exposures (Tremblay and Velasquez-Castro 2009). Therefore, at this time wind is not considered as the only potential factor to help determine the southern aspect of DCAs.

Lastly, skyline is also important for drones. In DCAs, open areas are extensive, yet urban or other visual markers are present at 10% of the cover, and the terrain is usually not steep (< 19% slope) (Figure 2). Tracks or other linear marks that may serve as landmarks in the orientation to find the DCAs and to return to the apiary are sparse (Figure 2). Urban cover is important to offer refuge for feral colonies that recolonize holes each year (Baum et al. 2008), but DCAs are also found in areas with limited urban cover.

Several physical factors were shown to be correlated with the occurrence of DCAs at candidate locations. If these features would have been known a priori, searching for areas without a southern aspect could have been avoided, allowing for a 100% improvement over our initial search criterion. Simple addition of slope (less than 20%) and urban zone cover (less than 10%) could have increased the positive rate to 75%. Puerto Rico, just as any other location, is likely to have particular characteristics; however, future comparison across DCAs in different places could help determine any potential hierarchy of landscape characteristics important for DCA formation. This first study on DCA landscape analysis could help finding these locations more reliably, in order to answer many additional research questions, such as behavioral correlates of DCA formation.

**Supplemental Figure.**
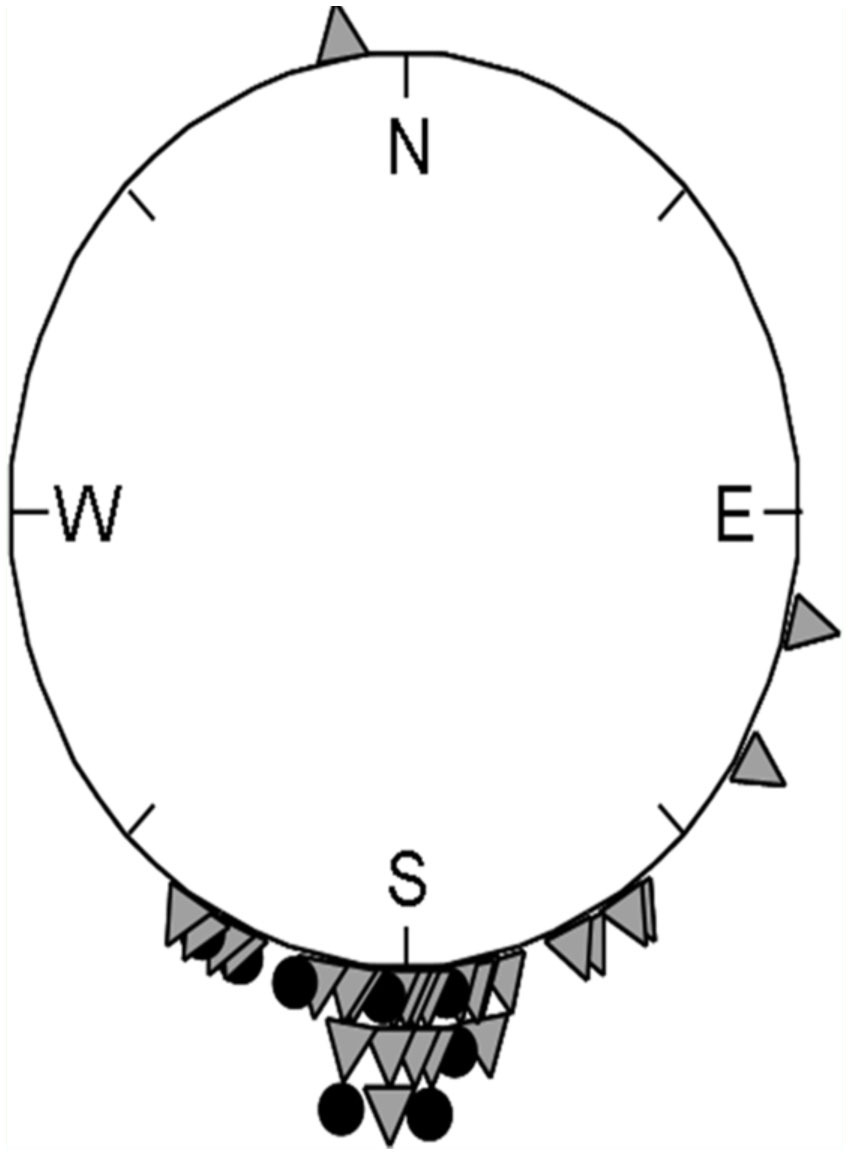
Representation of each buffer for the landscape analysis in each DCA and non-DCA. The buffers have radii of 100, 200, 400 and 800 meters. High quality figures are available online.

## Abbreviations

DCA(s),: drone congregation area(s);
DEM,: digital elevation model;
GIS,: geographical information system;
MRPP,: multi-response permutation procedure;
**RPA** =: recursive partitioning analysis
